# Naturalistic spatial education engages distinct neural processes for mental rotation, cognitive load, and linguistic demands

**DOI:** 10.1073/pnas.2601206123

**Published:** 2026-10-21

**Authors:** Eloise B. Gacetta, Marissa L. Laws, Lauren S. Aulet, Teoman S. Ozaydin, Jonathan S. Tsay, Conrad Zapanta, Jessica F. Cantlon

**Affiliations:** ^a^ Department of Psychology, Carnegie Mellon University, Pittsburgh, PA 15213; ^b^ Center for the Neural Basis of Cognition, Carnegie Mellon University and University of Pittsburgh, Pittsburgh, PA 15213; ^c^ Department of Psychological and Brain Sciences, University of Massachusetts Amherst, Amherst, MA 01003; ^d^ Neuroscience Institute, Carnegie Mellon University, Pittsburgh, PA 15213; ^e^ Department of Biomedical Engineering, Carnegie Mellon University, Pittsburgh, PA 15213

**Keywords:** fMRI, spatial cognition, parietal cortex, education, naturalistic paradigm

## Abstract

Neuroscience studies typically isolate spatial cognition using simplified tasks targeting a single cognitive process. However, during formal spatial education, the brain rarely performs one cognitive operation at a time. Here, we used functional magnetic resonance imaging (fMRI) to assess neural systems involved in dynamically processing an educational spatial visualization lesson. We quantified lesson content using an instructional-design framework capturing variation in spatial complexity, cognitive load, and verbal density. We then characterized how instructional demands modulated brain function using content-evoked responses, functional connectivity, and network-centrality. Spatial transformations during instruction robustly recruited parietal and premotor regions, which were linearly modulated by spatial complexity. This parietal dominance aligns with theories that these areas encode geometric and quantitative dimensions. In contrast, domain-general frontal activity tracked overall processing demands, and language regions tracked verbal density. Activity also varied with learners’ spatial abilities: higher spatial performers showed stronger modulation of parietal and premotor regions as spatial demands increased, and greater domain-general modulation across content types. Thus, the same circuitry representing spatial transformations during instruction also reflects individual variation in spatial cognition. Parietal regions showed the highest centrality and strongest within-network coherence, marking them as the central hub of the system, and distinguishing them from less central and less coherent domain-general regions. Together, the findings reveal how the brain dynamically decomposes complex spatial instruction, preserving parietal-centered spatial processing, while coordinating support from premotor, domain-general, and language systems. These findings begin to reveal how spatial cognition operates within the complexity of formal instruction.

## Significance

Spatial visualization skill is a foundational component of human intelligence and a powerful lever for success in science, technology, engineering, and mathematics (STEM). Yet little is known about how the human brain supports spatial reasoning during educational instruction. Using functional neuroimaging and a formal spatial visualization lesson in a large sample of 100 adults, we show that distinct dimensions of instructional content are selectively represented across neural systems. Parietal circuits centered on the intraparietal sulcus selectively tracked spatial transformations and scaled with learners’ spatial ability, demonstrating its central role during formal education. These findings help establish a neuroscientific framework for integrating educational research on spatial instruction with cognitive neuroscience.

Spatial intelligence is a cornerstone of human reasoning and a key predictor of achievement in science, technology, engineering, and mathematics (STEM). Spatial skills support decision-making and job performance in professions ranging from architect to mechanic to surgeon, and they predict entry and persistence in high-level STEM education (1–3). Critically, spatial abilities are highly trainable: targeted spatial interventions improve spatial visualization, mathematics, and geometry performance in both children and adults (3–8). In fact, a meta-analysis by Uttal et al. (9) revealed that spatial training yields robust improvements across tasks, ages, and genders, further highlighting the broad malleability of spatial skills.

One of the most successful educational implementations of spatial training is Sheryl Sorby’s Spatial Visualization Curriculum, initially developed for first-year engineering students at Michigan Technological University (10) but now is broadly available as a general education application (https://egrove.education/uses/spatial-visualization-training). We refer to this curriculum as “real-world” and “authentic” because the lessons are actually used to educate students in the real world through a digital app. The curriculum teaches students how to mentally manipulate and visualize three-dimensional forms through guided lessons, interactive exercises, and rotating-object demonstrations. Across multiple cohorts, Sorby’s curriculum has been shown to significantly improve spatial visualization abilities, increase performance in other STEM courses such as calculus and chemistry, and boost retention rates of women in engineering majors. This work demonstrates that explicit instruction in spatial visualization can produce far-reaching cognitive and academic benefits and provides a concrete educational framework for teaching it.

Spatial education is critical for human progress in science and technology. Uttal and Cohen (11) suggest that spatial skills may be a limiting factor for success in more challenging STEM classes. For example, Kozhevnikov et al. (12) found that individuals with low- and high-spatial skill interpret graphs differently, with high-spatial skill participants interpreting graphs with more ease. Integrating spatial interventions into formal education throughout development can substantially enhance foundations for success in academics and occupations and increase the talent pool for science and technology (8, 13–15). The present study investigates how the brain engages during formal spatial instruction in order to begin to understand the mechanics of spatial education.

The neural mechanisms engaged during instruction, where spatial content is embedded in a rich formal lesson, are largely unstudied. Instead, decades of studies have characterized the core cognitive and neural architecture of spatial visualization using controlled laboratory tasks. Foundational behavioral work by Shepard and Metzler (16) established mental rotation as a hallmark of spatial visualization by showing that the time required to judge whether two rotated objects match increases linearly with angular disparity. Neuroimaging studies by Kosslyn et al. (17, 18) demonstrated that these spatial transformations engage a distributed network of occipital, parietal, and premotor regions. The intraparietal sulcus (IPS) has emerged as a hub for spatial reasoning across mental rotation, mental arithmetic, and numerical reasoning (19–21), while premotor regions are engaged when spatial tasks involve imagined objects or movements (21). Lesion studies, neurodevelopmental disorders, and intracranial recording studies have shown that damage to the parietal lobe and premotor cortex causally disrupts spatial object manipulation, mental imagery, and action-related representations (22–27). Together, these findings indicate that parietal and premotor circuits jointly support object-centered transformations in space, providing a computational bridge between perception and action (28, 29).

Spatial reasoning also recruits domain-general control systems, including dorsolateral prefrontal (DLPFC) and anterior cingulate cortices (ACC) within the “multiple-demand network” (30, 31). However, the role of this network remains poorly defined: its constituent regions often vary across tasks, and their engagement may reflect distinct modulations by task difficulty or effort rather than a unified cognitive mechanism. Nonetheless, lesion studies indicate that damage to domain-general regions such as the DLPFC impairs fluid reasoning and visuospatial problem solving (32), suggesting that domain-general control can influence spatial performance. Although many demonstrations of domain-general or multiple-demand activity have used spatial and numerical tasks like spatial working memory, Raven’s matrices, arithmetic, N-back, or visuospatial reasoning paradigms, the key defining contrasts of the multiple-demand network are high- vs. low-load conditions. Load-sensitive functions may modulate neural activity in parietal and motor engagement during complex spatial learning. In contrast, the language network, which supports symbolic and verbal processes, is typically distinct from parietal–premotor systems and not involved in visuospatial reasoning (31). How these domain-general and linguistic systems interact with spatial circuits during real instruction when verbal, spatial, and reasoning demands naturally co-occur remains unknown.

During formal instruction, spatial learning unfolds through verbal content, images, demonstrations, cognitive control, and a host of other cognitive demands. Consequently, linguistic and domain-general circuits likely operate in concert with spatial circuitry–concurrent dynamics that are poorly captured by the isolated, nonecological tasks typically used in the lab. However, recent advances in “naturalistic” fMRI provide a framework for bridging this gap between the laboratory and education. Naturalistic fMRI paradigms capture neural responses during dynamic, ecologically valid experiences such as watching films or educational videos (e.g., refs. 33–38). Prior naturalistic studies show that functional networks can be reliably measured during complex input, and that their activity predicts functional distinctions observed in controlled tasks as well as individual learning outcomes. Importantly, naturalistic approaches are not simply more realistic, they are often essential for discovering the neurobiological origins of brain function, which emerge from interactions among multiple neural networks with distinct functional roles (34, 39, 40). Rigid experimental tasks, while precise, underestimate the richness of human cognition, whereas balancing rigor with ecological validity allows neuroscience to explain how the brain operates during real experiences (39). Naturalistic methods open the door to identifying how the brain integrates multiple information streams during instruction–such as during the educational experiences that improve spatial skills in the current study.

Only one prior fMRI study has tested neural changes related to spatial curricula (41, 42). That study measured neural activity before and after a year-long navigation and geospatial curriculum in 32 high school students–but it did not measure neural activity during the spatial instruction and did not find a relation between neural activity and mental rotation ability. Since mental rotation is the spatial ability with the highest predictive validity for academic and career achievement, it is critical to develop ways to track neural activity related to this ability during the spatial instruction itself. That is the focus of the current study.

To bridge the gap between cognitive neuroscience and spatial education, we applied fMRI to an educational video lesson from Sheryl Sorby’s Spatial Visualization Curriculum, an intervention that improves spatial reasoning in undergraduates (10), to measure how the brain tracks spatial content during instruction. The lesson introduces fundamental strategies for visualizing and mentally rotating three-dimensional forms. To quantify the information structure of this lesson, we developed a content-coding framework grounded in Cognitive Load Theory (43), a cornerstone of instructional design research. Each segment of the video was decomposed into cognitive units and coded along three theoretically motivated dimensions: rotational complexity (the number and dimensionality of object transformations), cognitive load (the cumulative visual, text, and novel conceptual information), and language density (the density of spoken and written words). Measures like these are used in the instructional design literature to estimate the processing demands of textbooks and lectures. We used these content coding methods in the current study to capture how spatial, cognitive, and verbal demands vary during the spatial visualization lesson.

Using fMRI data from a large sample of ~100 adults, we test how distinct neural networks track these content dimensions during formal spatial instruction. Building on prior evidence, we predict that parietal-premotor networks will track the rotational complexity of the lesson as learners mentally transform objects in space. In contrast, we expect that domain-general frontal-cingulate regions associated with cognitive control and working memory will scale with overall cognitive load, while language networks will track the density of verbal and textual information. Beyond these primary hypotheses, we also examine how individual differences in spatial skill relate to neural sensitivity across these networks, and how functional connectivity patterns among parietal, frontal, and language systems form the large-scale organization that supports spatial processing during formal instruction.

## Results

We analyzed fMRI data from students viewing a spatial education lesson from a broader curriculum that successfully improved their spatial visualization abilities (*SI Appendix*, Fig. S5). Thus, participants improved over the broader curriculum from which the scanned lesson was drawn. Our goal was to determine how neural networks encode the interacting spatial, cognitive, and linguistic demands of a spatial lesson. In Results, we first define the task-based regions of interest (ROI) and functional networks that form the basis for all subsequent analyses. Next, we test how neural activity within those networks varies with the instructional content of the lesson. We then assess how individual differences in spatial ability modulate these neural responses. Finally, we examine network functional connectivity and centrality to test how these networks are organized during spatial instruction.

### Defining ROI and Functional Networks.

**Fig. 1.**
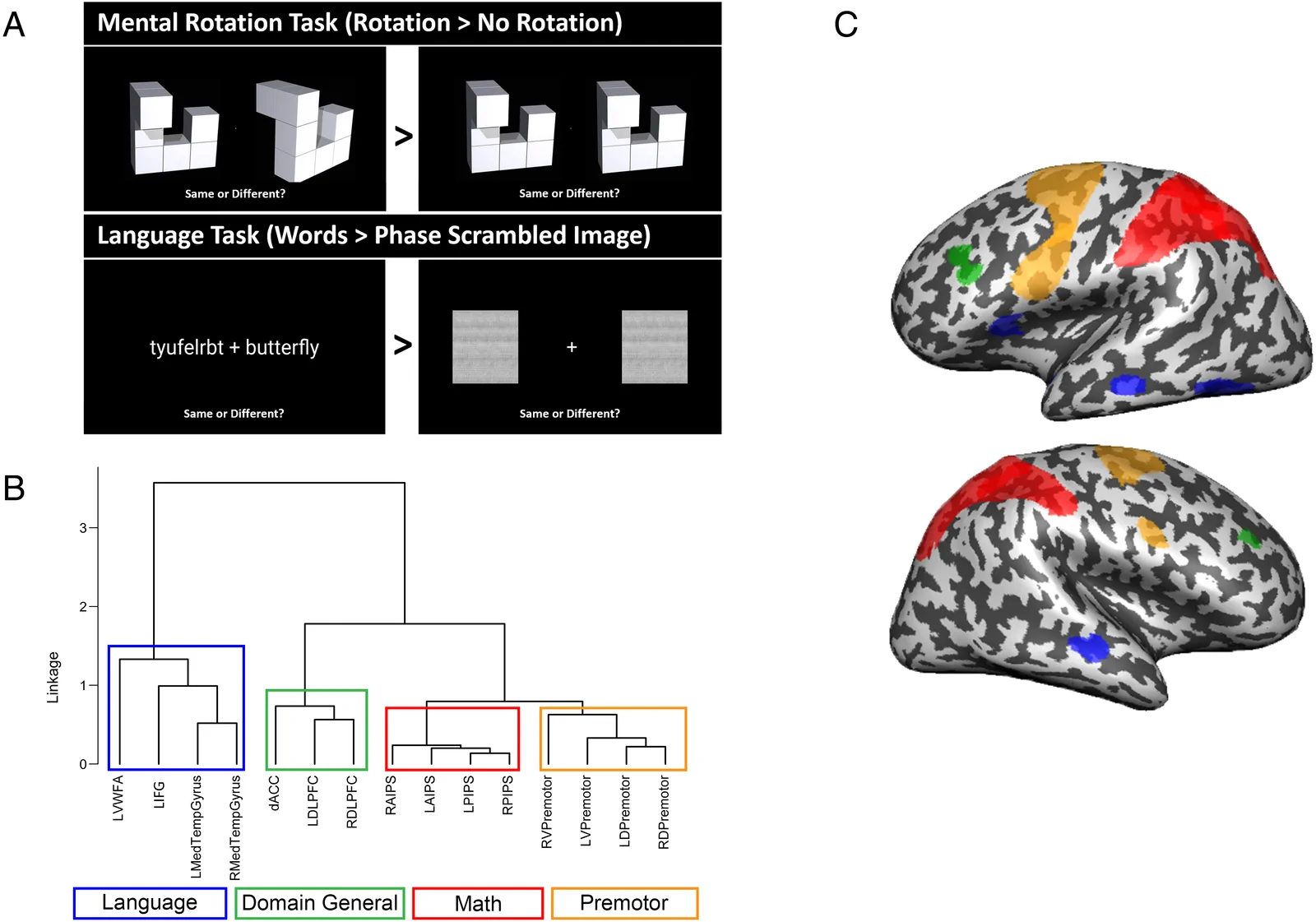
Experimental tasks for ROI definition, functional network organization, and cortical localization of ROIs. (*A*) Task schematics for the mental rotation spatial localizer (Rotation > No Rotation) and language localizers (Words > Phase-Scrambled Image) used to identify ROIs. Participants judged whether a pair of stimuli were the same or different. (*B*) Hierarchical clustering of ROIs based on functional connectivity similarity derived from *N* = 89 participants. The dendrogram shows Ward’s hierarchical clustering applied to Euclidean distances computed from ROI connectivity feature vectors, with an additional penalty applied to cross-task ROI pairs. Colored boxes indicate the functional network groupings identified in the clustering solution. Branch height reflects dissimilarity between ROIs, with lower linkage heights indicating greater similarity in connectivity profiles. Colored boxes denote the four resulting functional networks: Language (blue), Domain-General (green), Math (red), Premotor (orange). (*C*) Surface projections of the identified functional networks on the cortical surface. Colors correspond to the network assignments shown in panel *B*.

We identified ROIs to serve as anchors for our analyses using theory-driven cognitive tasks. A mental rotation localizer task (Rotation > No Rotation) and a language localizer (Words > Phase Scrambled) identified 15 ROIs used for network-based analyses (Fig. 1*A*). These ROIs were thus defined independently of the educational lesson and were selected based on their a priori cognitive functions. We then used the lesson data in a separate second step to characterize how these independently defined ROIs were neurally related during spatial instruction. Specifically, we applied a semisupervised hierarchical clustering analysis to functional connectivity among the ROI neural timecourses from the educational lesson. This procedure was used to identify network organization among independently localized regions, not to define the ROIs themselves. These analyses thus characterize the connectivity structure among independently localized ROIs during instruction and are not intended as an independent validation of canonical large-scale networks.

In our analysis, adding a modest task penalty to the clustering procedure improved the alignment of cluster structure (penalty = 0.5, ARI = 1.0). This clustering analysis yielded four coherent networks that align with established functions in cognitive neuroscience (Fig. 1*B*). The Math network included bilateral anterior and posterior IPS, regions consistently implicated in spatial visualization, numerical processing, and object-based transformations. The Premotor network encompassed premotor regions of the bilateral dorsal and ventral premotor cortex, reflecting systems involved in planning and simulating object manipulation. The Domain-General network comprised the dorsal anterior cingulate cortex (dACC) and bilateral dorsolateral prefrontal cortex (DLPFC), regions associated with cognitive control and task demand. Finally, the Language network consisted of the left visual word form area (VWFA), left inferior frontal gyrus (IFG), and bilateral middle and superior temporal gyri, consistent with regions supporting orthographic, phonological, and semantic processing. The resulting organization from this data-driven clustering algorithm reproduces classic distinctions among networks. And, despite sharing the same localizer origin, Math, Premotor, and Domain-General ROIs segregated into distinct branches.

This two-step approach allowed us to identify regions independently using cognitive localizers and then characterize their functional networks using separate data. We use these independently localized ROIs and their network labels in subsequent analyses.

### Content-Based Neural Responses during Educational Lesson.

To test whether distinct neural systems track the cognitive demands of instruction, we modeled the content of the spatial education video using a quantitative framework from the multimedia learning and education literature (43). This approach treats instructional materials as streams of information that vary in their processing demands and those varying demands have consequences for learning. Each segment of the lesson was coded along three theoretically grounded dimensions derived from Cognitive Load Theory (43): 1) Rotational Complexity (number and dimensionality of spatial transformations), 2) Cognitive Load (weighted sum of presence/absence of new spatial concepts, text, dynamic elements, and rotational complexity), and 3) Linguistic Density (spoken + written words). These measures quantify how spatial, cognitive load, and verbal demands fluctuate throughout the lesson, allowing us to test whether specific neural networks are tuned to particular instructional content.

**Fig. 2.**
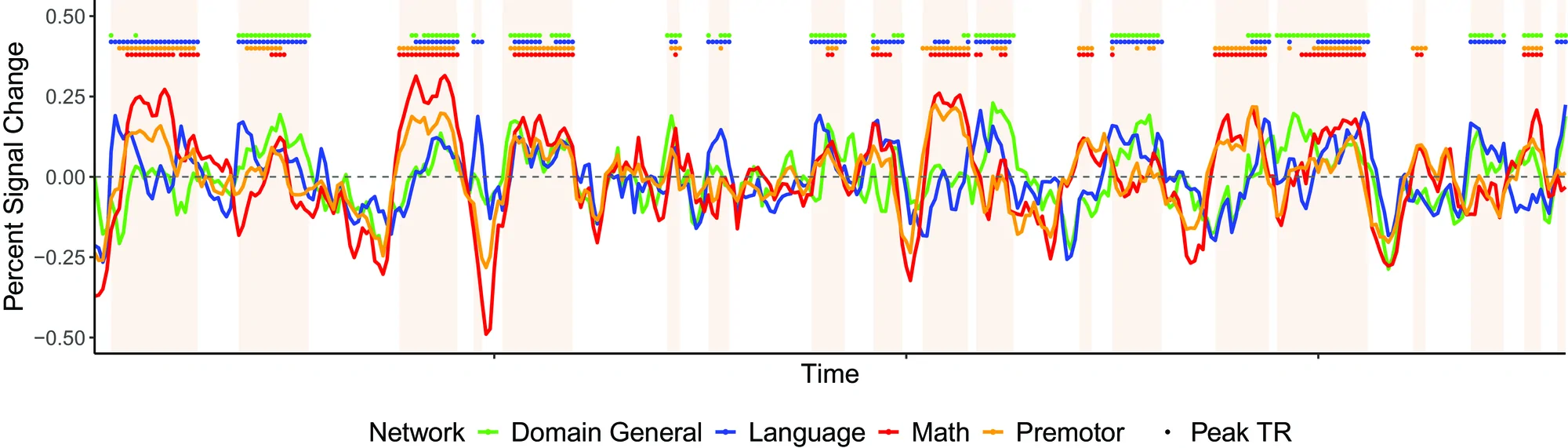
Reverse correlation neural events. Group-averaged mean-normalized timecourses for each functional network across the entire video lesson. Colored traces represent mean PSC within each network at each TR: Domain-General (green), Language (blue), Math (red), and Premotor (orange). Tan-shaded regions denote neural events included in analyses, identified with peaks ≥ 0.75, 1, and 1.25 SD of signal change for each ROI. Colored dots above the traces indicate TRs included in neural events for each network, with dot color corresponding to the associated network.

We identified significant neural events corresponding to peaks in the group BOLD timecourses from each network. Peaks were defined as BOLD activity exceeding thresholds of 0.75 SD, 1 SD, and 1.25 SD above the mean signal (Fig. 2). We then used a linear mixed-effects model to predict mean-normalized percent signal change (PSC) from Network (Math, Premotor, Language, Domain-General) and the three content dimensions (Rotation Complexity, Cognitive Load, and Linguistic Density) with a random effect of subject, identifying the unique variance attributed to each instructional predictor over and above others.

**Fig. 3.**
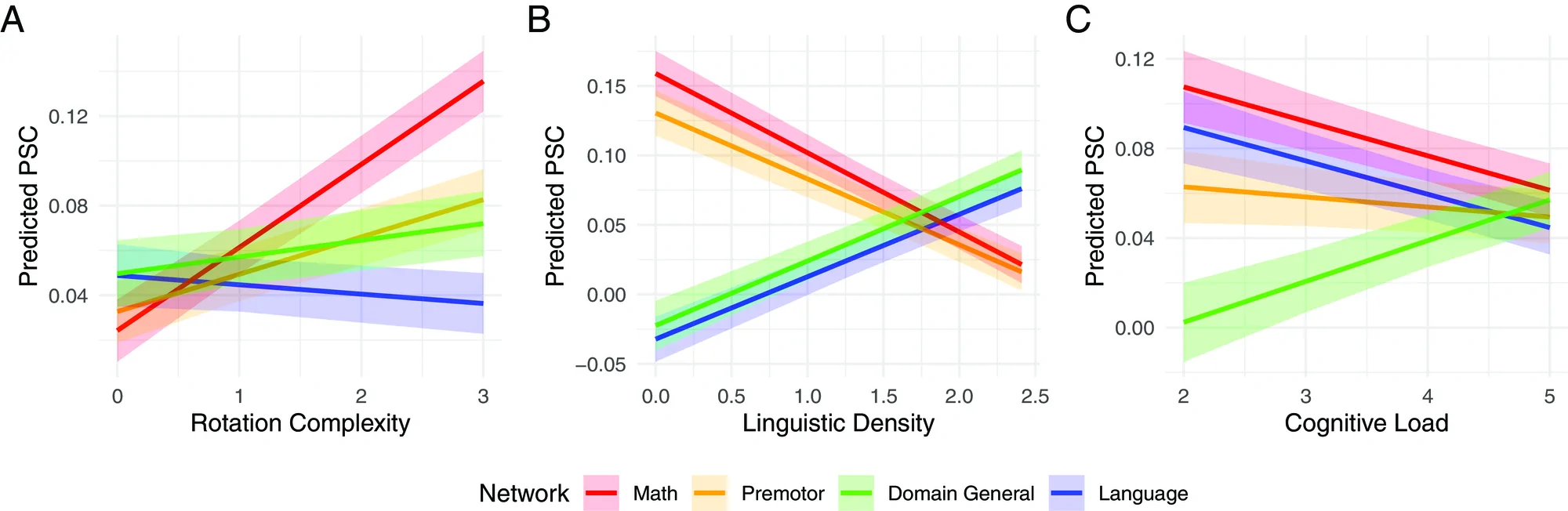
Predicted PSC across functional networks as a function of instructional content. Panels show model-estimated mean-normalized PSC activity from the linear mixed-effects model for (*A*) Rotation Complexity, (*B*) Linguistic Density, and (*C*) Cognitive Load across Math, Premotor, Domain-General, and Language networks. Solid lines represent predicted values and shaded ribbons indicate 95% CI. Predictions were derived from the full interaction model including all instructional content–network interactions, with participant included as a random intercept (*N* = 89 participants).

Relative to the Language network, mean-normalized activity was significantly higher in the Math network (β = 0.14, *t* = 5.54, *P* < 0.001), centered on the IPS, lower in the Domain-General network (β = −0.19, *t* = −6.62, *P* < 0.001), and did not differ in the Premotor network (β = 0.032, *t* = 1.22, *P* = 0.22) throughout the video lesson. Critically, these networks diverged according to the content of the educational video. Fig. 3 shows that the three content types, Rotation Complexity, Linguistic Density, and Cognitive Load, elicited different responses in networks. Rotation Complexity positively modulated activity in the Math (β = 0.037, *P* < 0.001), Premotor (β = 0.017, *P* < 0.001), and Domain-General networks (β = 0.007, *P* = 0.009), but not the Language network (β = −0.004, *P* = 0.092). A significant Math network × Rotation Complexity interaction (β = 0.043, *t* = 8.90, *P* < 0.001) and a significant Premotor network x Rotation Complexity interaction (β = 0.020, *t* = 4.01, *P* < 0.001) reflects that rotation complexity drove increased activation in the Math network and Premotor network more positively than in the Domain-General network (β = 0.012, *t* = 2.21, *P* = 0.027) and Language network (Fig. 3*A*). In contrast, Cognitive Load positively modulated the Domain-General network (β = 0.018, *P* < 0.001), whereas activity decreased in the Math (β = −0.015, *P* < 0.001) and Language networks (β = −0.015, *P* < 0.001) and did not reliably change in the Premotor network (β = −0.004, *P* = 0.084). Relative to the Language network, Cognitive Load had a significant strong positive activation in the Domain-General network (β = 0.039, *t* = 7.11, *P* < 0.001), as well a weaker but positive significant activation in the Premotor network (β = 0.017, *t* = 3.32, *P* < 0.001), whereas the Math network did not differ (β = −0.0036, *t* = −0.66, *P* = 0.51) (Fig. 3*C*). Linguistic Density positively modulated the Language (β = 0.045, *P* < 0.001) and Domain-General networks (β = 0.047, *P* < 0.001) but negatively modulated the Math (β = −0.057, *P* < 0.001) and Premotor networks (β = −0.075, *P* < 0.001). Relative to the Language network, Linguistic Density drove significant negative effects in the Math network (β = −0.092, *t* = −13.02, *P* < 0.001) and Premotor network (β = −0.077, *t* = −10.88, *P* < 0.001) and did not differ from Domain-General network activation (β = 0.0031, *t* = 0.40, *P* = 0.69) (Fig. 3*B*).

Together, these results reveal a clear functional dissociation: parietal-premotor regions of the Math and Premotor networks selectively track spatial transformations, the Domain-General network is modulated by all three types of demand, and the Language network is selectively modulated by verbal content.

### Spatial Skills Relate to Neural Activation during Instruction.

Individual differences in performance offer insights into which differences in neural activity during spatial instruction are related to spatial skills. As shown in the previous section, the Math and Premotor networks only showed positive effects of Rotation Complexity (not Linguistic or Cognitive Load); the Language network only showed positive effects of Linguistic Density (not Rotation or Cognitive Load); and the Domain-General network showed positive effects for all three content types. Within these effects, we next tested whether high and low spatial skill groups differed in either a main effect on neural activity or in their slopes of neural activity. Brain–behavior relationships were also analyzed using continuous measurements of spatial skill (*SI Appendix*, Fig. S4).

**Fig. 4.**
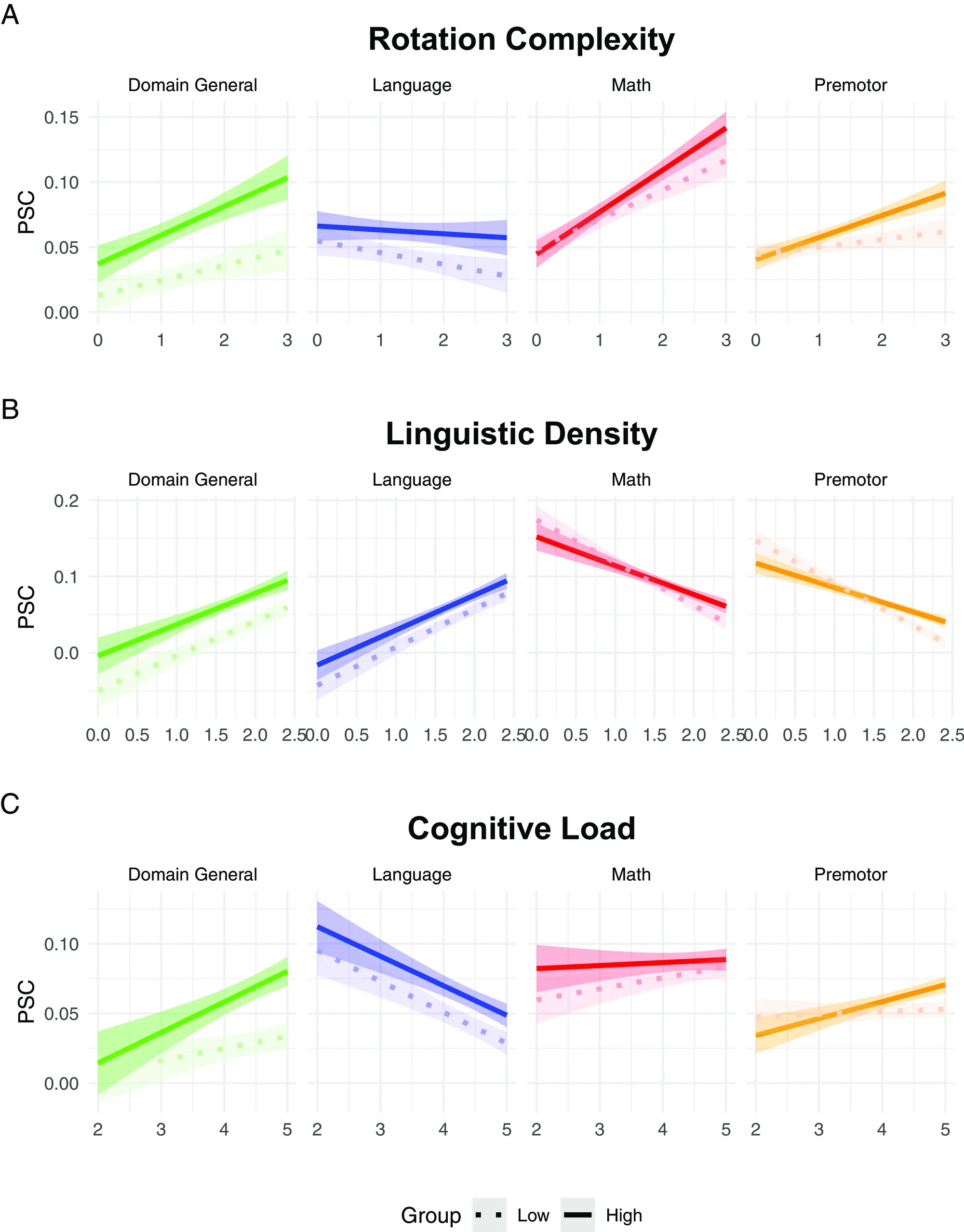
PSC in each functional network in response to instructional content across spatial skill groups. Panels depict the relationships between PSC and (*A*) Rotation Complexity, (*B*) Linguistic Density, and (*C*) Cognitive Load within the Domain-General, Language, Math, and Premotor networks. Separate lines are shown for High (solid, *n* = 45) and Low (dotted, *n* = 42) spatial skill groups. Shaded regions represent 95% CI around the fitted regression lines. Colors denote network identity, with lighter shading indicating the Low group and darker shading indicating the High group. These results hold when spatial skill is entered as a continuous variable rather than categorical (*SI Appendix*, Fig. S4) and when neural events are defined at 0.75, 1.0, and 1.25 SD thresholds (*SI Appendix*, Fig. S2).

To examine the brain–behavior relationships, we compared neural activity between high- (*n* = 45) and low-skill (*n* = 42) participant groups across the three instructional content dimensions. Fig. 4 shows the results of a mixed-effects regression predicting neural activity (mean-normalized PSC) from Group, Network, Cognitive Load, Rotation Complexity, and Linguistic Density (with random intercepts for subject). High- and low-skill participants exhibited qualitatively similar patterns of content-specific neural responses. In both groups, Math and Premotor networks increased with Rotation Complexity and decreased with Linguistic Density; the Language network increased with Linguistic Density and decreased with Cognitive Load; and the Domain-General network increased across all three content dimensions. This correspondence between two independent groups is noteworthy because it demonstrates that the content-based neural signatures predicted by our hypotheses and prior literature were robust and reproducible across individuals with different levels of spatial skill. However, there were quantitative differences between groups in overall amplitude of activation and in the slope of activation across content demands.

**Fig. 5.**
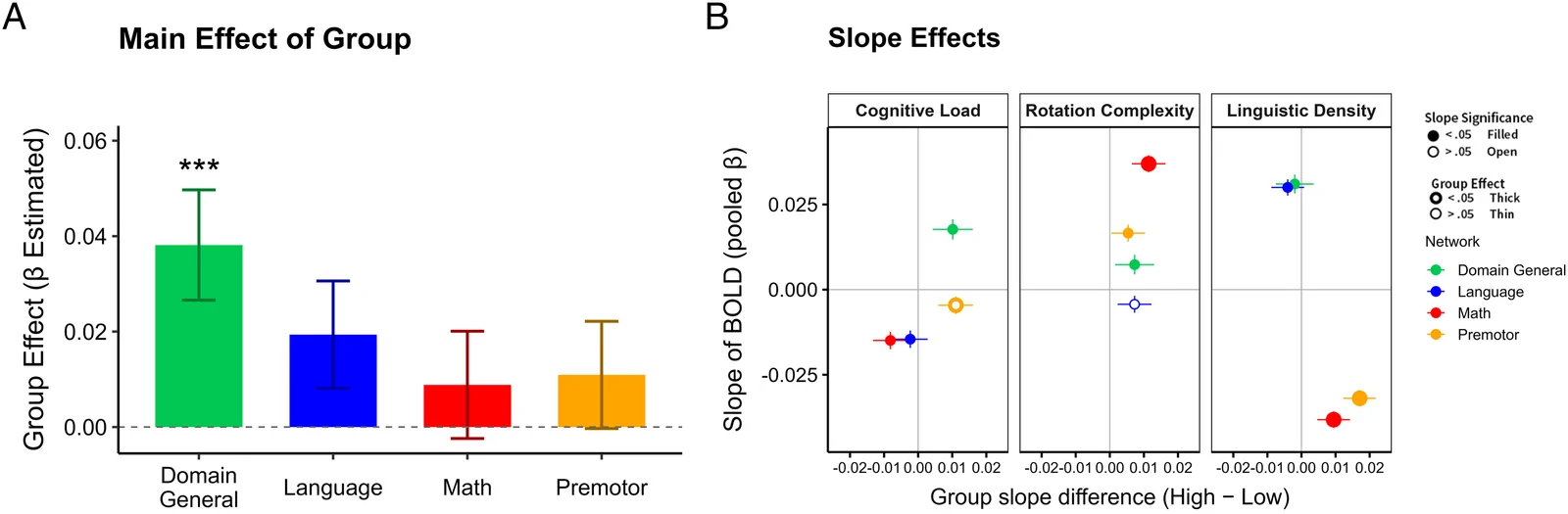
Group effects on mean-normalized PSC across functional networks and instructional content dimensions derived from the full linear mixed-effects model (*N* = 87 participants). (*A*) Main effect of group (High−Low) across Domain-General, Language, Math, and Premotor networks. Bars represent estimated β coefficients and error bars indicate ±1 SE. The asterisks denote a statistically significant group effect (*P* < 0.001). (*B*) Slope effects for Cognitive Load, Rotation Complexity, and Linguistic Density across functional networks. The *x*-axis represents the estimated group slope difference (High−Low), whereas the *y*-axis represents the pooled neural activity slope across groups. Colored points indicate network identity. Filled circles denote significant pooled slope effects (*P* < 0.05), whereas open circles indicate nonsignificant pooled effects. Thick outlines indicate significant group differences in slope (*P* < 0.05), whereas thin outlines indicate nonsignificant group differences. Error bars represent ±1 SE for both pooled slope estimates and group slope differences.

First, we quantified how the amplitude of neural responses differed between high- and low-skill groups by testing for main effects of Group. Across content dimensions, we found a reliable main effect of Group in the Domain-General network: high-skill participants showed greater neural responses than low-skill participants for all content types in this network (High−Low: β = 0.038, *z* = 3.30, *P* < 0.001), whereas main effects of Group in the Language, Math, or Premotor networks were smaller and did not reach significance (all |*z*| ≤ 1.72, *P* ≥ 0.08; Fig. 5*A*). Thus, domain-general frontal regions were more engaged in high-skill participants than low-skill participants across all content demands.

We then asked whether the slopes of the content functions differed between groups (Fig. 5*B*). Fig. 5*B* plots pooled slope values from positive to negative on the y-axis and the degree of group differences in slopes on the x-axis to show which networks were positively modulated by a content demand and also showed group differences (upper right quadrant). For Rotation Complexity, slopes were positive across groups in the Math, Premotor, and Domain-General networks, but only the Math network showed a significant group difference in slope: high-skill participants exhibited a steeper increase in neural activity with Rotation Complexity than low-skill participants (High−Low: β = 0.011, *z* = 2.30, *P* < 0.05), consistent with stronger rotational tuning in parietal math regions. Linguistic Density produced positive slopes in the Language and Domain-General networks, but there were no group differences in those slopes. Finally, the only network with a strong positive pooled slope for Cognitive Load was the Domain-General network, but its slope did not differ reliably between groups (High−Low: β = 0.010, *z* = 1.72, *P* = 0.08)–indicating that high- and low-skill participants similarly ramped up domain-general activity as tasks became more demanding. Thus, the Math network in the Rotation Complexity condition showed group differences in slopes, while other networks showed more uniform group differences or nonsignificant patterns of content-based modulation across skill levels. Consistent effects were obtained when spatial ability was modeled continuously, demonstrating that the group differences were not an artifact of dichotomizing the spatial-ability measure (*SI Appendix*, Fig. S4).

### Functional Connectivity Varies between Networks.

To characterize the functional connectivity profiles of the networks during spatial instruction, we examined functional connectivity among the 15 independently localized ROIs across the full lesson timecourse. The clustering analysis described above assigned ROIs to Math, Premotor, Domain-General, and Language networks; the present analysis then quantified the magnitude of connectivity within each identified network and between each pair of networks. These analyses thus are not an independent test of the network labels but rather quantify how strongly each network was internally coherent and how strongly it interacted with other networks during instruction.

**Fig. 6.**
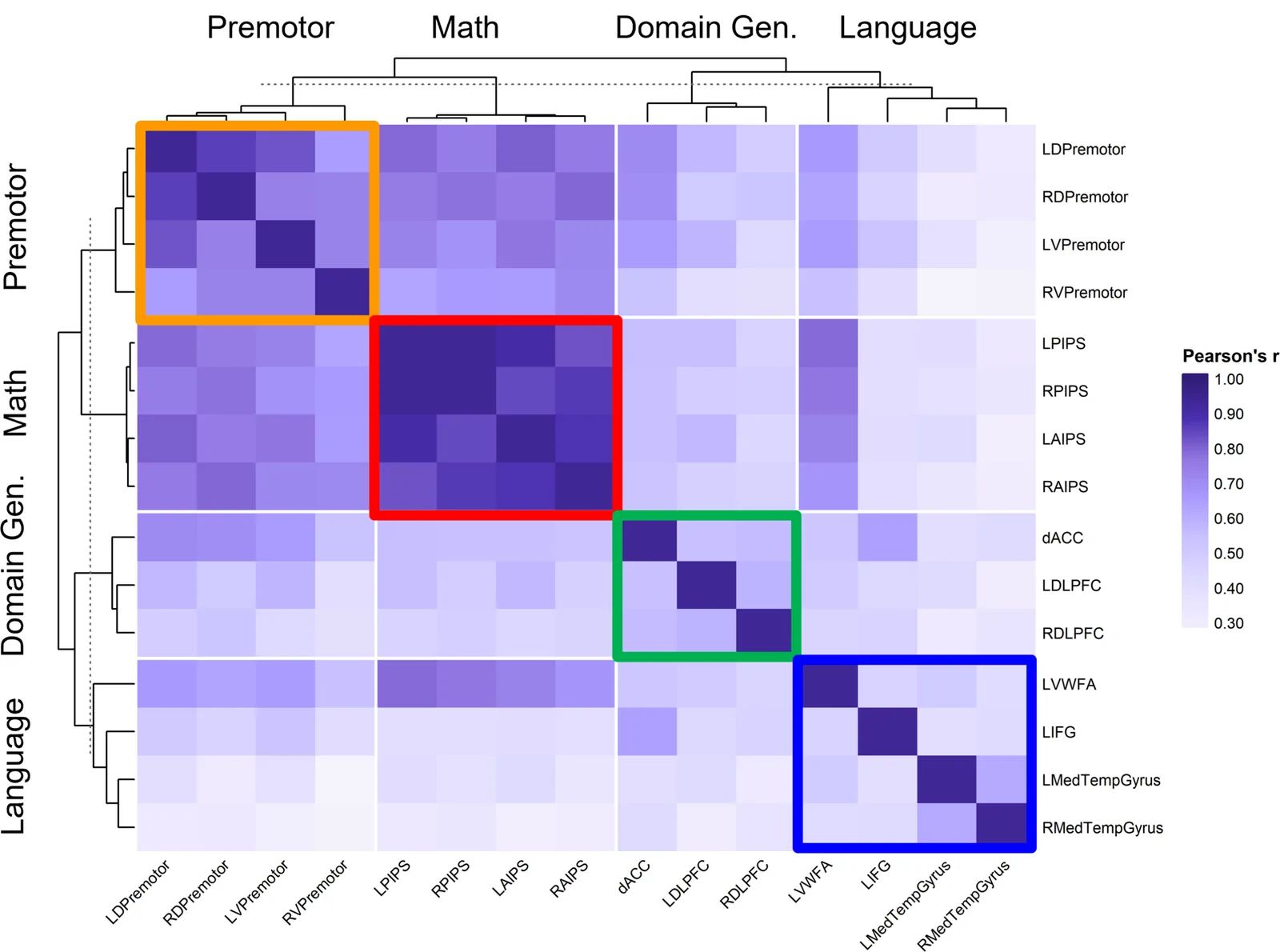
Group-level functional connectivity matrix across ROIs from *N* = 89 participants. Heatmap values represent Pearson correlation coefficients (r) between ROI time series converted from fisher transformed z-scores, with darker colors indicating stronger functional connectivity. Dendrograms along the rows and columns represent the network membership of the corresponding ROI. Colored boxes highlight within-network connectivity structure: Premotor = orange, Math = red, Domain-General = green, Language = blue.

A Pearson’s r heatmap of functional connectivity revealed especially high correlation between Math and Premotor networks during the educational video (Fig. 6), suggesting that parietal and premotor systems form a coordinated circuit during spatial learning. To quantify these interactions, we conducted a linear mixed effects analysis (*N* = 89) with a random intercept for Subject on within-network and between-network connectivity, calculated by averaging together the connectivity of each ROI to every other ROI in the same or different network. Within-network connectivity was positive and significant for each network: Language (β = 0.51, *t* = 22.74, *P* < 0.001), Math (β = 0.87, *t* = 43.83, *P* < 0.001), Premotor (β = 0.50, *t* = 24.95, *P* < 0.001), and Domain-General (β = 0.13, *t* = 6.50, *P* < 0.001), confirming that the network definitions reflected coherent functional systems.

Between-network functional connectivity provided evidence of the integrative role of parietal and premotor connectivity in spatial cognition. Math and Premotor networks showed strong positive coupling (β = 0.43, *t* = 21.702, *P* < 0.001), consistent with the hypothesis that parietal IPS-centered circuits interface with premotor systems to support object-based spatial visualization. The Math network also coupled positively, though more weakly, with Domain-General regions (β = 0.050, *t* = 2.50, *P* = 0.013), suggesting that parietal engagement is partially modulated by general reasoning demands. Similarly, the Premotor network showed positive connectivity with the Domain-General network (β = 0.10, *t* = 5.19, *P* < 0.001). By contrast, the Language network and Domain-General network showed marginally significant negative connectivity (β = −0.040, *t* = −2.00, *P* = 0.045), and the Language network showed no significant functional connectivity with the Math or Premotor networks, highlighting the separation of verbal-symbolic and visuospatial-motor circuits during spatial learning.

These results support previous research suggesting that both parietal regions (Math network) and premotor regions (Premotor network) work together during spatial processing (21). Together, these findings align with our hypotheses that parietal and premotor systems form an integrated network during spatial visualization, and that domain-general regions contribute more peripherally. The absence of connectivity with language regions underscores the functional specificity of this parietal-premotor circuit for spatial processing.

### Left IPS Has High Centrality.

**Fig. 7.**
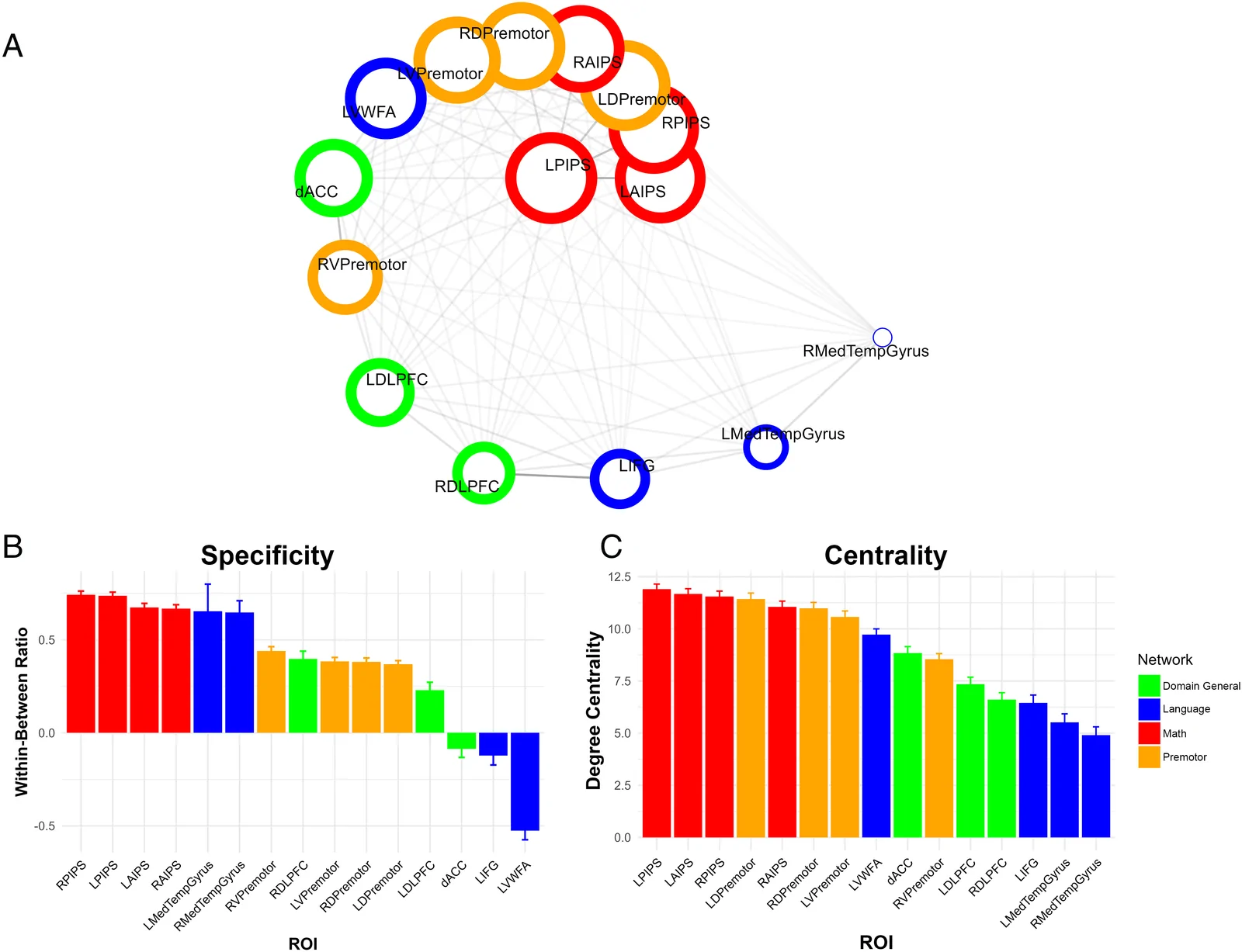
Degree centrality of ROIs. Colored by network: Premotor = orange, Math = red, Domain-General = green, Language = blue. (*A*) Degree centrality and participation coefficient. The center represents the highest degree centrality hub (left posterior IPS), the distance to the center represents the closeness to the hub, the width of the ring represents participation coefficient, and the edges represent topological similarity. (*B*) Bar graph of log-transformed within–between ratio per ROI ± SE. (*C*) Bar graph of degree centrality values per ROI ± SE.

To characterize the role of each region within the functional network, we computed degree centrality to quantify how strongly each ROI was connected to all other nodes, and within–between network connection ratios to assess the diversity of each ROI’s connections across networks. The degree centrality analysis revealed that both anterior and posterior left IPS regions exhibited the strongest overall connectivity (Fig. 7*C*). Across networks, parietal and premotor regions showed highest degree strengths, indicating that they serve as major hubs supporting parietal-premotor integration during spatial instruction. Regions within the Domain-General network displayed moderate degree strength, consistent with a role in integrating information across multiple systems. In contrast, Language network regions exhibited substantially lower degree strength, suggesting that this network remained functionally segregated from the others during the video.

Within–between ratios of node connections measured the extent to which an ROI is specialized within its own network or is a more generalized connector to all other networks (Fig. 7*B*). Bilateral posterior IPS had the strongest within-network specificity (right = 0.742, left = 0.737), followed by the bilateral anterior IPS (right = 0.668, left = 0.674). Further, bilateral MTG demonstrated high within–between ratios (right = 0.653, left = 0.648). The regions that showed the lowest within–between ratio included the left IFG (−0.112), dACC (−0.0855), and left DLPFC (0.223). Thus, during spatial instruction, IPS regions combined high centrality with high network specificity: they were strongly embedded in the Math network while occupying the most central positions in the broader functional network. The Language and Domain-General networks were, by comparison, less central and less specialized.

At the ROI level, the left IPS exhibited slightly higher degree centrality than the right IPS, while posterior IPS regions in both hemispheres showed higher participation coefficients than their anterior counterparts. These findings suggest that left intraparietal regions are somewhat more dominant during spatial processing than the right, whereas the posterior IPS regions in both hemispheres play a greater role in linking the Math network to other systems than anterior regions. These results are broadly consistent with meta-analyses reporting bilateral parietal engagement during spatial and mathematical tasks (19) but extend prior work by showing that parietal subregions vary in how centrally they are embedded within the larger functional network during spatial instruction.

Finally, within the Language network, the VWFA demonstrated comparatively high degree centrality. Reading orthographic material and processing complex line junctions are known to activate the VWFA (44, 45). Thus, VWFA engagement may reflect the text processing and other structured visual demands of the lesson.

## Discussion

The spatial education lesson elicited broad neural responses spanning domain-general and language networks, but the spatial components of the lesson were dominated by a parietal-premotor circuit centered on the IPS. Activity within this network scaled selectively and parametrically with spatial transformation demands, formed the strongest and most cohesive functional network during the lesson, and varied systematically with learners’ spatial abilities. In contrast, language and domain-general frontal systems tracked verbal and general processing demands more broadly but did not show the same degree of spatial selectivity or network centrality. This pattern suggests that formal spatial education recruits the same neural circuits that underlie intuitive spatial cognition in controlled tasks and provides initial links between human neuroscience and real-world educational interventions. As we discuss below, these findings bear on how instructional content shapes neural processing, why learners differ in spatial performance, and how parietal systems support complex spatial and mathematical reasoning.

To bridge human neuroscience with educational research, we used insights from Cognitive Load Theory (Sweller et al., 1988) which proposes that instructional materials vary in the extent to which they consume limited cognitive resources. Cognitive Load Theory is a widely used and domain-general framework in educational science. It has been applied across mathematics, science, engineering, reading comprehension, medical education, and other domains of instruction to characterize how instructional materials affect the cognition and therefore the learning of students. A central prediction of the theory is that different instructional demands place distinct pressures on representational, linguistic, and executive systems involved in learning. The theory distinguishes among intrinsic load, which reflects the inherent complexity of the material; germane load, which refers to the cognitive effort devoted to building task-relevant representations; and extraneous load, which arises from irrelevant or poorly organized information that unnecessarily taxes cognitive processing. Effective instruction is achieved when extraneous load is minimized, allowing learners to devote more capacity to intrinsic and germane processing.

One contribution of the current study is that it identifies neural mechanisms associated with these distinct forms of instructional demand during an educational lesson. Rotational Complexity selectively recruited parietal-premotor systems associated with spatial representation and transformation, consistent with the role of germane load in constructing task-relevant representations. In contrast, the text-dense elements of the lesson reflect extraneous load, because coordinating linguistic descriptions with the spatial elements of the lesson introduces additional processing demands. These demands increased activation in the Language network and the Domain-General network while suppressing Math and Premotor activity, suggesting that learners reallocated resources toward linguistic processing and away from spatial systems when language complexity rose. Finally, the general cognitive effort required to integrate multiple pieces of information during learning was reflected in increased activation of the Domain-General system across all conditions (rotation, language, and general cognitive load), consistent with its role in managing total cognitive demand. Thus, our data show how different instructional loads relate to neural responses in distinct neural systems, offering mechanistic support for Cognitive Load Theory principles. These findings could help establish a broader framework for connecting educational theory with the neural processing of instructional content, and for validating instructional loads using converging behavioral and neural measures (46, 47).

Another important contribution of the present study is that it reveals how individual differences in spatial skill can emerge during instruction itself. Although high- and low-spatial skill learners showed qualitatively similar patterns of content-specific neural responses, two key differences emerged. First, high-skill learners showed greater Domain-General activation across the lesson than low-skill learners, suggesting greater engagement of cognitive control resources during instruction overall. Second, increases in rotational complexity produced significantly stronger modulation of the spatially specialized Math network in high-skill learners. This group-specific slope effect only appeared in the spatially specialized Math network, where high-skill learners showed stronger engagement when the lesson required complex transformations of spatial representations. Thus, while high-skill learners may exhibit broader advantages in domain-general engagement, the spatial differences appear when instruction requires increasingly complex spatial representations. These findings highlight that individual differences in spatial skill are not confined to performance outcomes but emerge during the instruction process itself. A key prediction is that improvements in spatial skill should be accompanied by broader engagement of domain-general systems during instruction and selective recruitment of parietal systems during complex spatial transformations, a hypothesis that will require longitudinal data.

Our results also provide evidence that the IPS is at the center of spatial and quantitative reasoning. Several theories propose that parietal circuits encode spatial and mathematical properties, particularly analog dimensions such as size, length, angle, number, distance, and orientation (e.g., refs. 48–52). Relatedly, Ayzenberg and Behrmann (29) identified the functions of the parietal cortex in size-, view-, and rotation-invariant object representations, which are spatial computations. In the present study, the Math network centered on IPS showed scaling with Rotation Complexity rather than with cognitive or linguistic load, indicating that this circuit encodes transformations of objects in space rather than nonspecific task difficulty as others have argued (30, 31). Degree centrality results revealed the left IPS as the primary hub, followed by the right IPS and then premotor regions. Importantly, this finding that left IPS is central to spatial processing during instruction converges with prior findings from Cortes et al. (41), which identified the left IPS as a critical region for learning from a spatial curriculum. In comparison to the parietal cortex, Language and Domain-General regions showed more distal and distinct network architecture. Together, these results from a naturalistic spatial visualization lesson indicate that the IPS implements computations that are central to the spatial visualization of objects, even during high-level spatial instruction, and thus may underlie the transfer from intuitive spatial reasoning to formal mathematical learning (20, 49, 53).

These findings additionally bear on debates concerning whether spatial and executive systems form a unified multiple-demand architecture or function as dissociable networks (30). Our results show distinct functional profiles for the Math and Premotor networks compared to the Domain-General and Language networks. In the hierarchical clustering analysis, the Domain-General network emerged as its own separate cluster even though Math, Premotor, and Domain-General ROIs come from the same localizer (so the task definition cannot be the driver of the observed differences). In follow-up network analyses, the Domain-General network also showed much lower within-network connectivity (β ≈ 0.13) than Math (β ≈ 0.87) and Premotor (β ≈ 0.50), indicating less internal coherence. The Math network contained the strongest hubs in the system, with IPS regions showing both high overall centrality and strong within-network connectivity. The parietal hubs were closely coupled with Premotor regions, forming a cohesive circuit. In contrast, Domain-General fronto-cingulate regions showed weaker internal coherence and lower centrality, together with relatively distributed cross-network connectivity, consistent with a supporting rather than central role. These network-level dissociations were mirrored functionally: the Domain-General network was most strongly related to general cognitive load, whereas the Math network was preferentially tuned to rotation complexity, indicating a distinct functional tuning to spatial content. Together, these findings argue against an undifferentiated frontal-parietal “multiple-demand” architecture (30, 31) and instead support a functionally specialized architecture in which domain-general and spatial circuits play dissociable roles.

Finally, our findings have implications for understanding the neural mechanics of how spatial instruction translates into educational outcomes. Decades of research demonstrates that spatial skills are malleable and that training in spatial visualization yields improvements in mathematics, STEM coursework, and long-term academic outcomes (3, 9, 10). The present results extend this educational literature by identifying the neural mechanisms that are engaged when students encounter spatial visualization lessons. Parietal and premotor systems not only track spatial content during instruction but also reflect variation in students’ spatial skills. These circuits have been implicated in intuitive, analog spatial transformations in prior work, and the present findings show that the same systems are engaged when students encounter technical spatial relations in an instructional context (20, 49, 51, 54). In this way, the present findings connect human neuroscience research on parietal involvement in spatial, mathematical, and mental rotation tasks with the educational processes through which students engage spatial cognition during formal instruction. Although our approach substantially increases ecological validity relative to traditional neuroscience tasks and captures the digital learning environment for which this curriculum was designed, it does not capture the full social complexity of the classroom. Future work should therefore test how these neural dynamics generalize across educational settings, change over the course of learning, and how they may inform evidence-based educational practice.

## Materials and Methods

### Ethics.

All experiments were approved by the Carnegie Mellon University Institutional Review Board (Protocol #STUDY2023_00000324). Written informed consent was obtained from all participants prior to participation. The study was determined to pose no more than minimal risk, and all data were coded to protect confidentiality. Participants were compensated with course credit or monetary payment.

### Participants.

A total of 117 adults participated in the study (Female = 61%, mean age ± SD = 23.74 ± 5.71). Participants were recruited through the Carnegie Mellon University Psychology Department research pool, psychology and engineering courses, and flyers. Courses included Professor Zapanta’s capstone engineering course and Changing Your Brain, a humanities course for undergraduates. Some participants were excluded from certain analyses due to excessive motion (5 mm), drowsiness during the task, preprocessing issues, or incomplete behavioral data as described below.

### Behavioral Measure.

Prior to fMRI testing, participants were administered the paper-and-pencil Purdue Spatial Visualization Test: Rotations (PSVT:R) (55, 56) according to standardized instructions as a measure of spatial ability (10). The test included 30 multiple-choice questions framed as analogies, asking participants to apply the spatial transformation shown in the example object to a new object and select the correct answer from five options (*SI Appendix*, Fig. S1). Raw scores (out of 30) were used in analyses. A binomial threshold identified participants performing at or below chance [≤10/30 correct, binomial test, *P* > 0.05, R function qbinom(0.95, size = 30, prob = 0.2) + 1]. Two participants were excluded on this basis.

### fMRI Stimuli.

#### Naturalistic educational video.

All participants viewed an 11:52-min educational video titled Rotation About One Axis from Sorby’s Developing Spatial Thinking curriculum (10). This curriculum is widely used for training spatial skills in undergraduate STEM students, and shows evidence of effectiveness, transfer, and predictive validity. In the video, participants watched a teacher-led explanation of the principles of object rotations and axes of rotations, were introduced to and taught the “right-hand rule” and “arrow coding,” and practiced visualizing rotations. The teacher then used her hands as a physical representation of the rotation of 3D objects and drew the final result of the transformation. Objects were composed of 5 to 14 cube blocks in a variety of configurations and were rotated 90, 180, or 270 degrees from the original orientation of the object. Participants were instructed to watch attentively. The full video is available in *SI Appendix*.

#### Mental rotation localizer.

We used an updated version of the Shepard and Metzler (16) mental rotation task to localize ROIs engaged during spatial visualization (57) in a sample of 100 adults. In each trial, two 7-block 3D block objects were simultaneously presented for 4 s and participants had to determine whether the two shapes were the same or different. The objects had three conditions at varying angles (0 degrees, 100 degrees, 150 degrees, Fig. 1*A*). The contrast Rotation > No Rotation was used to localize regions related to spatial processing (parietal, premotor, domain-general regions).

#### Language localizer.

We included the language network as a control to provide a comparison system not expected to be engaged by spatial visualization. ROIs for the language network were drawn from prior literature identifying regions that respond selectively to linguistic content (58, 59). These included classic perisylvian areas such as left IFG (−48, 16, 24), left middle temporal gyrus (−55, −18, −13), right middle temporal gyrus (55, −14, −13), and VWFA (−43, −54, −12). Because these regions are already well established, we relied primarily on the published literature for their localization and used a small subset of participants from the current study (*N* = 13) to validate them with an in-house language localizer. Three categories of stimuli were presented: English words, Navajo words, and phase-scrambled images of the English words. Participants completed a discrimination task in which they judged whether pairs of words were same/different. Each pair contained one intact word and either a word with validly rearranged letters, or a word with invalidly rearranged letters. Phase-scrambled images were presented in the same manner, with pairs being either identical or different (Fig. 1*B*). Each run consisted of 12 blocks and 6 trials per block for a total of 72 trials, with each pair of stimuli presented every 2 s followed by 2 s of blank screen. The contrast Words > Phase Scrambled Words was used to localize language-related regions, which aligned with the established ROIs.

### Data Processing and Statistical Analysis.

fMRI acquisition, preprocessing, and software for the following analyses are described in *SI Appendix*, *Supplementary Methods*.

#### ROI and network definition.

The ROI were defined at the group level from independent localizer tasks, and networks were labeled independently, from the lesson data, based on their connectivity. The analysis proceeded in two separate steps:

1. ROI Definition (Localizer Task Data). Parietal, premotor, domain-general, and language regions were defined from two localizer tasks, a language task and a mental rotation task, independent of the lesson.
2. Network characterization (Lesson Timecourses). We labeled those independently defined regions as network clusters using separate data. Specifically, we used a data-driven hierarchical clustering of their functional connectivity to determine how regions group together during the lesson. This step is used to group ROIs into coherent clusters for peak activation analysis, not to define the ROIs themselves.

A total of 15 ROIs were identified across both localizer tasks. Four language-related regions were identified using a general linear model with a contrast of Words > Phase Scrambled Words (GLM; *P* < 0.05): bilateral medial temporal gyrus, left-lateralized IFGs, and the left-lateralized VWFA. Eleven spatial regions were identified using a GLM contrast of Rotation > No Rotation (*N* = 100; *P* < 0.001): bilateral anterior IPS, bilateral posterior IPS, bilateral dorsal premotor cortex, bilateral ventral premotor cortex, bilateral DLPFC, and dACC. We used the Rotation > No Rotation contrast because it enabled us to capture spatially sensitive neural regions as well as domain-general regions to comprehensively test neural function. Coordinates for ROIs are shown in *SI Appendix*, Table S1.

For network definition, we then measured subjects’ BOLD activity from each ROI during the whole naturalistic video lesson. A semisupervised hierarchical cluster analysis was conducted to identify subnetworks among the 15 ROIs. For each subject, we extracted BOLD time series from each ROI during the naturalistic video lesson and computed ROI–ROI Pearson correlations, which were Fisher z-transformed and then averaged across subjects to obtain a group-level connectivity matrix. To quantify distance between regions, those matrices were converted to Euclidean distance matrices, such that highly correlated ROIs were close together and weakly correlated ROIs were farther apart. Hierarchical clustering was then performed on this distance matrix using Ward’s linkage, which iteratively merges ROI clusters to minimize within-cluster variance (SSE) and produce compact, internally coherent groupings. To respect the broad task boundary between language and spatial ROIs, a penalty was added to cross-task linkages (language vs. spatial) when computing distances. We evaluated task penalties using the Adjusted Rand Index (ARI). The optimal language vs. spatial penalty was determined empirically by testing a range of values and identifying the smallest penalty that produced an ARI of 1, which was +0.5 (60). No penalties were imposed within the mental rotation set of 11 ROIs, allowing subnetworks to emerge naturally from the clustering. See *SI Appendix*, Table S1 for further ROI details. The resulting dendrogram contained coherent Math, Premotor, Domain-General, and Language branches, which we refer to as “networks” because of their empirical definition by a data-driven functional connectivity analysis.

#### Reverse correlation analysis of neural events.

A reverse correlation analysis was used to define neural events, and relate those events to lesson content, following methods from Hasson et al. (36) and Richardson et al. (38).

First, we calculated mean-normalized PSC for the entire lesson timecourse. PSC was calculated at the individual ROI level from each participant’s raw signal for each volume by mean-normalizing (dividing each volume’s intensity value by the mean of the entire time course, multiplying by 100, and subtracting 100). Group mean PSC was then calculated for each volume. Then, each volume was shifted by two TRs to align PSC with the actual video moment and account for the hemodynamic lag. Next, neural events were derived using a peak detection approach applied to the group-averaged timecourse. Peaks were defined as BOLD responses 1 SD above the mean signal for each ROI. Breaks of 1 to 2 volumes within peaks were treated as part of the same event; however, breaks of ≥3 volumes were considered a new event, as ~6 s of hemodynamic lag had passed. For each ROI, we retained the top 60 volumes exceeding the 1 SD threshold. This prevented any one ROI from disproportionately determining the event set. In total, 148 unique lesson volumes were included in the union of neural events across ROIs, with a total duration of 4:24 min across 17 discrete blocks, amounting to 37% of the timecourse. For robustness, we also analyzed the data with events defined by 0.75 SD and 1.25 SD thresholds, and the results are consistent with our main results at a threshold of 1 SD (*SI Appendix*, Fig. S2).

Next, we separately coded the amount of linguistic content, rotational complexity, and overall content in the video from the slides and transcript. The linguistic content was quantified as the total number of spoken syllables and written on-screen characters, summed and log-transformed to normalize distribution (range = 0 to 2.44). The complexity of rotations was coded for the degree of rotation being illustrated: 0 = no rotation; 1 = single-axis 90° rotation; 2 = multiaxis rotation or right-hand-rule operation; 3+ = compound multiaxis rotation sequences. Cognitive load was estimated as the overall amount of information from text + narration + graphics + mental exercises. We used an operationalization consistent with cognitive load theory from the multimedia instruction literature, which emphasizes that the working memory burden during learning depends on the number of interacting elements that must be processed simultaneously (43, 61–63). We adopted a simplified content-based code that maps onto Sweller’s principle of element interactivity (43), where element interactivity is defined as the number of interacting information elements that belong to the learning task. Our three coding levels reflect increasing element interactivity: Level 1 is isolated items of information (e.g., term definitions, concepts, and labels) within one mode (e.g., either text or picture) and low element interactivity, Level 2 is integration of both verbal and visual elements (e.g., text + diagrams + demonstrations) requiring coordination of multiple formats, and Level 3 is multistep manipulations such as mental exercises or practice where several elements must be represented and maintained simultaneously. These values were summed to capture total load imposed by simultaneous demands. For consistency, annotation was supported by a large language model applied to transcripts and slides, with all tags validated with humans.

Finally, we used the instructional content codes as parametric predictors of neural peaks in regression analyses. Mixed effects models were conducted for each ROI and each volume within an event: PSC ~ Network * (Rotation Complexity + Linguistic Density + Cognitive Load) + (1/Subject). Therefore, each volume for each ROI within a network was taken into account in the model. Correlations among the content types during the lesson were low (*SI Appendix*, Fig. S3) and all variance inflation factors were well below threshold in regression analyses, indicating no problematic collinearity (*SI Appendix*, Table S2).

#### Functional connectivity.

We calculated pairwise ROI Pearson’s r correlation analyses for individual participants and Fisher transformed (Fisher’s z). We then averaged each individual z-scored connectivity matrix across participants and converted back to r values for interpretation. Within-network connectivity was the mean of all Fisher-transformed pairwise correlations among ROIs within a network. Between-network connectivity was the mean of all Fisher-transformed pairwise correlations of an ROI from one network to the ROI of the other network, excluding duplicated values from the matrix. A mixed-effects model predicting X ~ Y included all between- and within-network connectivity values to control for all other networks and individual subject differences.

#### Degree centrality, participation coefficient, and within–between connection ratio.

We used a graph theory network analysis to quantify the role of each ROI within functional networks using individual z-score matrices. For each ROI, degree centrality (node strength) was the sum of its positive connection strengths with all other nodes, computed using the igraph (64) and brainGraph (65) packages in R. Z-scores were thresholded at 0.25 to ensure only meaningful connections were included. Regions with higher degree centrality were interpreted as hubs, as they demonstrate overall greater influence. To characterize the extent to which each ROI connected across multiple functional networks, we computed weighted participation coefficients using brainGraph. Values approaching 0 indicate that a node’s connections are concentrated within a single network, whereas higher values indicate a more distributed pattern of connectivity and therefore interpreted as connector hubs linking multiple functional systems. To quantify network specialization of each ROI, we computed within–between network connectivity ratios for each ROI and participant. For each ROI, we computed the log-transformed mean positive connectivity to ROIs within the same functional network and to ROIs in other networks, normalizing each by the number of connections. A small constant (1.0 × 10^−6^) was added to the numerator and denominator to ensure numerical stability. Larger positive within–between ratio values reflect a stronger within-network specialization, whereas lower values reflect more distributed, cross-network connections.

#### Brain–behavior.

To calculate relationships between network activation and spatial skill ability, participants were split into two groups (Low and High performers) using a median split (*M* = 23) based on their baseline PSVT:R score following methods of previous educational studies examining low and high performers (10, 66). PSVT:R scores were mean centered before conducting statistical analyses with PSC and cognitive content (*N* = 87). Individual PSC values were used for analyses that tested PSC predicted by Group, content, and network. Original brain–behavior models tested the interaction between Group and content while controlling for those predictors and a random effect of subject. Three-way interaction models between group, content, and network were tested with a similar model that included network as a predictor variable. Group effects were calculated with a direct contrast of High−Low performers from the regression models.

## Supplementary Material

Appendix 01 (PDF)

Movie S1. Spatial visualization lesson. Developing Spatial Thinking curriculum (13).

## Data Availability

Anonymized fMRI data have been deposited in KiltHub (https://doi.org/10.1184/R1/30898949) (67).
