# Supplementary material for: Naturalistic spatial education engages distinct neural processes for mental rotation, cognitive load, and linguistic demands
Source: Proc Natl Acad Sci U S A. 2026 Oct 21;123(43):e2601206123. doi: 10.1073/pnas.2601206123 (PMC13715823; doi:10.1073/pnas.2601206123)
Supplement: Supplementary file 1 [file pnas.2601206123.sapp.pdf]

Supporting Information for
Naturalistic Spatial Education Engages Distinct Neural Processes for Mental Rotation, Cognitive Load, and Linguistic Demands

Eloise B. Gacetta, Marissa L. Laws, Lauren S. Aulet, Teoman S. Ozaydin, Jonathan S. Tsay, Conrad Zapanta, & Jessica F. Cantlon

Eloise B. Gacetta, Jessica F. Cantlon
Email: egacetta@andrew.cmu.edu, jcantlon@andrew.cmu.edu

This PDF file includes:

Figures S1 to S6
Tables S1 to S2
Legends for Movies S1
SI References

Other supporting materials for this manuscript include the following:

Movies S1

Supplementary Methods

fMRI Acquisition

Whole-brain BOLD imaging was conducted on a 3T Siemens MAGNETOM Prisma scanner with a 32-channel head coil at the CMU-Pitt Brain Imaging Data Generation and Education Center (RRID:SCR_023356). High resolution T1-weighted anatomical images were acquired using a magnetization-prepared rapid gradient echo sequence (MPRAGE) (TR = 2500 ms, TE = 2.9 ms, flip angle = 8 degrees, voxel size = 1.0 × 1.0 × 1.0 mm isotropic, FOV = 256 × 256 mm, matrix size = 256 × 256, 176 interleaved sagittal slices with anterior–posterior phase encoding, slice thickness = 1.0 mm with no gap, GRAPPA acceleration factor = 2) at the beginning of each session. Functional images were collected using a T2*-weighted echo-planar imaging sequence (TR = 2000 ms, TE = 30 ms, flip angle = 79 degrees, voxel size = 2.0 × 2.0 × 2.0 mm isotropic, FOV = 212 × 212 mm (matrix size = 106 × 106), 72 interleaved transverse slices with anterior–posterior phase encoding, multiband acceleration factor = 3) and a total of 360 volumes were acquired in a single functional run.

fMRI Preprocessing

Functional MRI data were preprocessed using BrainVoyager QX 22.4 (1) and a custom Python-based script pipeline that included: (1) DICOM-to-BV file conversion, (2) slice-timing correction using acquisition-specific slice tables and cubic spline interpolation, (3) 3D motion correction using six motion parameters via sinc interpolation, with all volumes aligned to the first functional run of the session, (4) spatial smoothing (6 mm FWHM Gaussian kernel), and (5) temporal high-pass filtering with three iterations of sinc interpolation. Following preprocessing, the functional volumes were aligned to the first volume of the first run of the session, which was coregistered to the T1-weighted MPRAGE anatomical image using automatic initial alignment and manual final alignment in BrainVoyager. The structural images were manually converted to the ACPC plane and Talairach space (2) using anatomical landmarks. Transformation files from each step were then applied to convert the preprocessed functional data to Talairach space using sinc interpolation at a 2 × 2 × 2 mm resolution.

Software

Preprocessing and analysis of structural and functional MRI data was carried out in BrainVoyager 22.4 for Windows. MATLAB 2024a was used to extract the raw timecourse signal fMRI data from BrainVoyager and computing fisher-transformed group average correlation matrices and individual subject's ROI pairwise correlation matrix using the Neuroelf toolbox v.1.1 (http://neuroelf.net) and the BVQX package, and was used to create protocol and single-subject design matrix files. Brain figures were visualized in BrainVoyager 22.4 for Windows. Statistical analyses were performed in R Statistical Software via RStudio (3). Mixed-effects models were run via the lme4 package and lmerTest package (4, 5), ARI analyses were run via the mclust package (6). Figures from statistical analyses were visualized using the ggplot2 package (7) and ComplexHeatmap package (8). Degree centrality and participation coefficient were performed through the igraph package (9) and brainGraph package (10) in R.

Figures

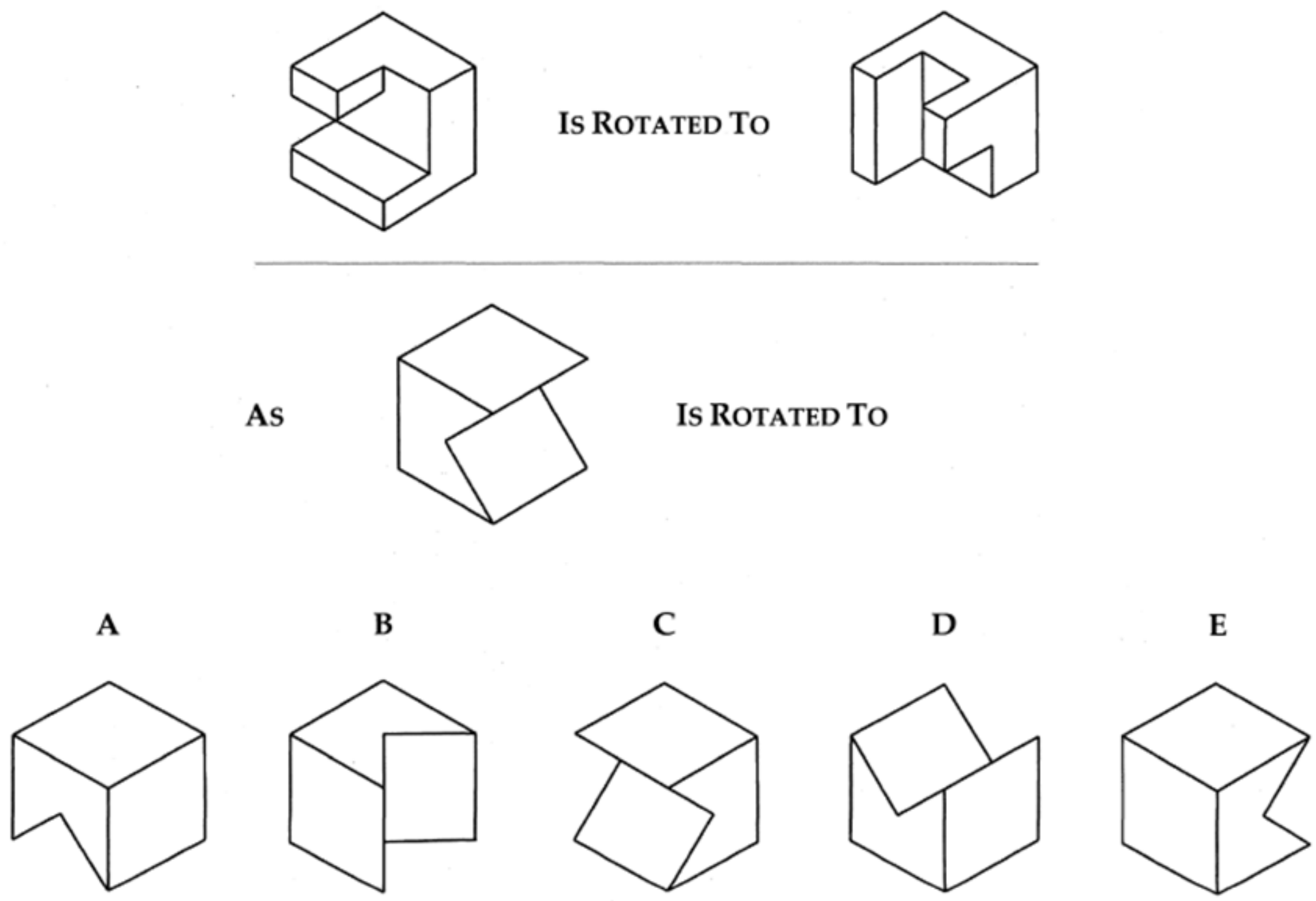

Fig. S1. Example question of the baseline spatial skill assessment Purdue Spatial Visualization Test: Rotations. The Purdue Spatial Visualization Test: Rotations (11, 12) is a 30 multiple-choice pen-and-paper 3-D mental rotation assessment that requires participants to identify the rotated version of the target object. Participants completed this assessment prior to the MRI, and spatial skill groups were defined on a median split such that a score greater than or equal to a score of 23 classified a high performer (*n* = 45), and less than 23 classified a low performer (*n* = 42). This is following the methods of previous literature on spatial education (13).

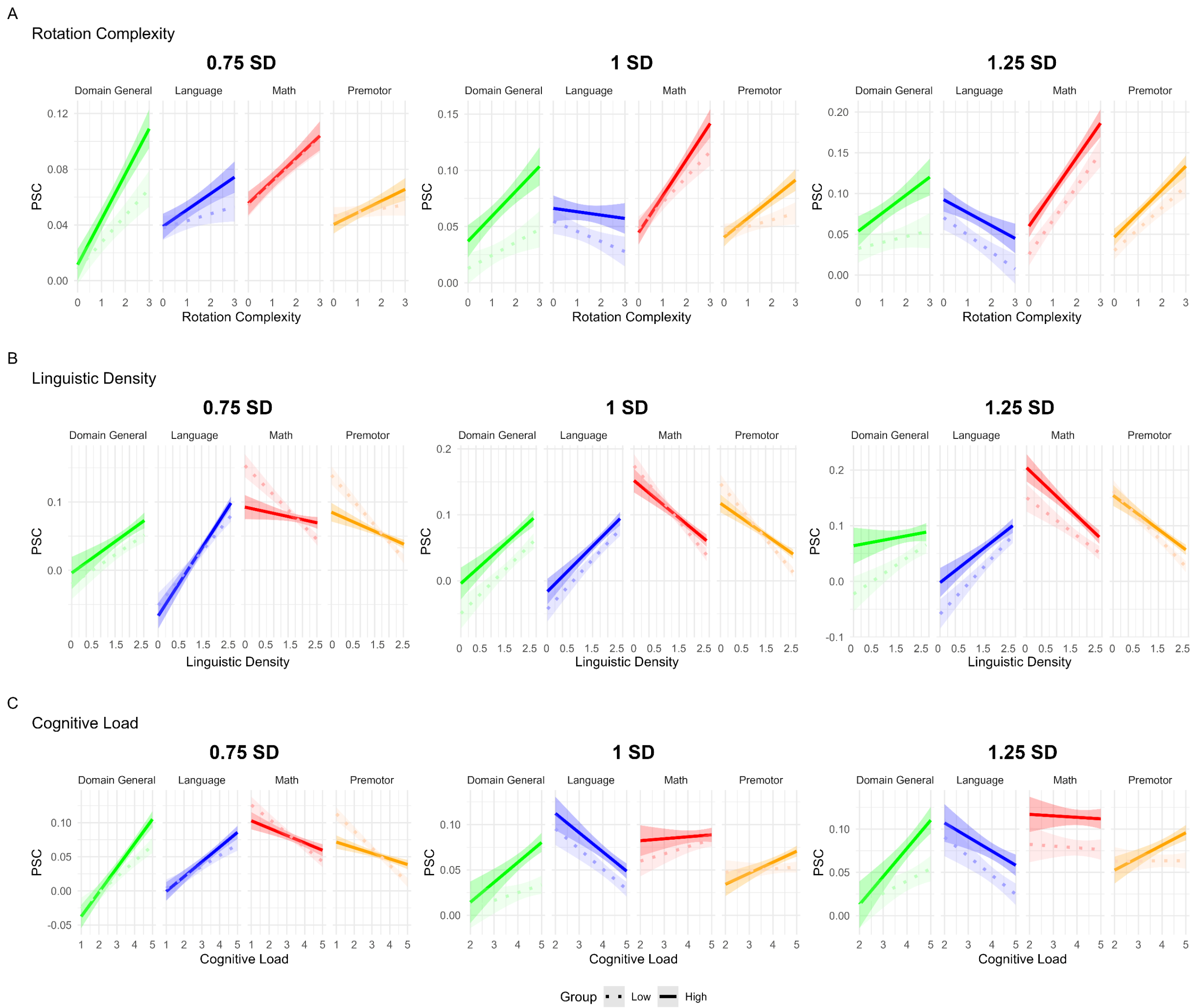

Fig. S2. Event-parsing results across alternative event boundaries defined using PSC thresholds of 0.75 SD, 1 SD, and 1.25 SD greater than the mean PSC. Panels show the relationship between PSC and instructional content dimensions across the four functional networks. Shaded ribbons indicate confidence intervals around the model-estimated trends. The overall pattern of neural activation remained consistent across all threshold definitions, demonstrating the robustness of the reported effects to variation in event-boundary criteria.

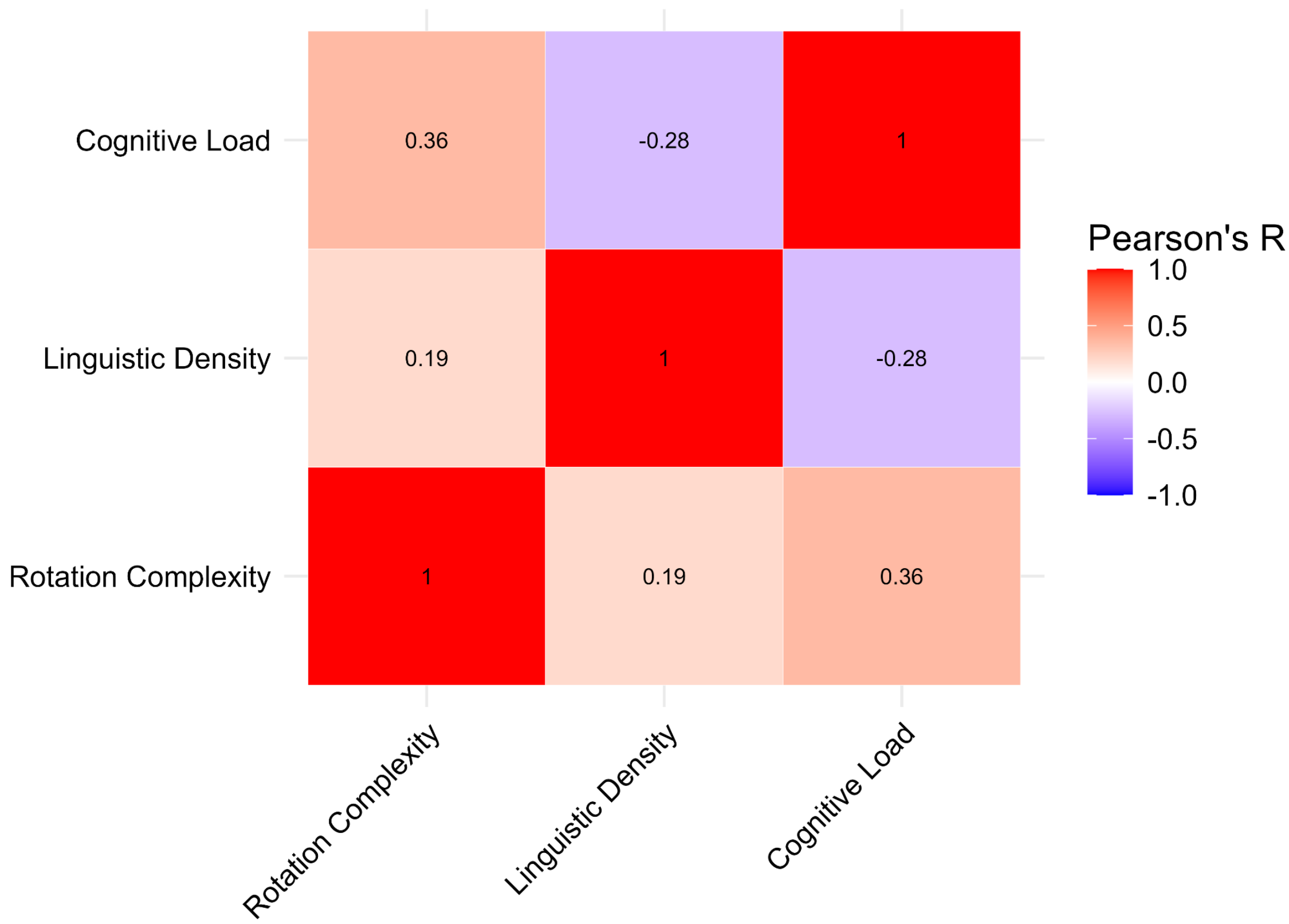

Fig. S3. Correlation matrix of instructional load variables during the video lesson: Rotation Complexity, Linguistic Density, and Cognitive Load. Heatmap values represent Pearson correlation coefficients (r) calculated across instructional loads for neural events. Numerical values within each cell denote the corresponding Pearson correlation coefficient. All r values are non-significant, demonstrating relative independence among instructional variables.

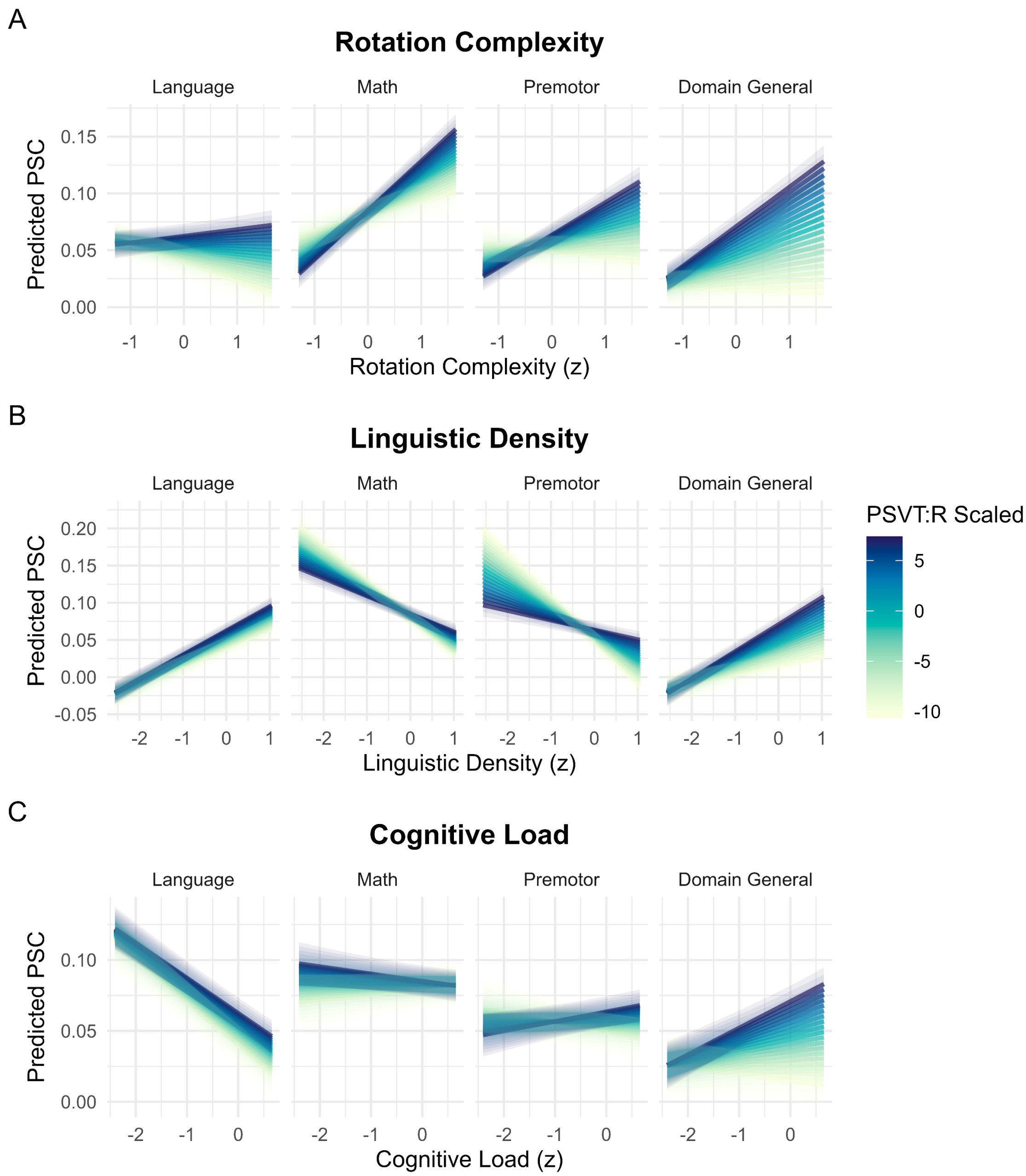

Fig. S4. Predicted PSC as a function of instructional demands with a continuous PSVT:R scale (*N* = 87) across functional networks. Panels show fixed-effect predictions from linear mixed-effects models examining interactions among PSVT:R score as a centered continuous variable and network for (A) Rotation Complexity, (B) Linguistic Density, and (C) Cognitive Load. Line color represents PSVT:R scored (scaled) with darker colors indicating higher spatial visualization ability. Shaded ribbons indicate ±1 SE around model predictions. All predictors were z-scored prior to analysis, and models included participant as a random intercept while controlling for the remaining instructional content dimensions. The categorical analysis reported in the main text was designated as the primary analysis because it directly tests the educationally motivated question of whether instructional demands differentially engage neural systems in groups with lower versus higher spatial ability. The continuous analysis presented here provides a complementary robustness test, demonstrating that these brain–behavior relationships also emerge across the full range of spatial ability and are not an artifact of dichotomizing PSVT:R scores.

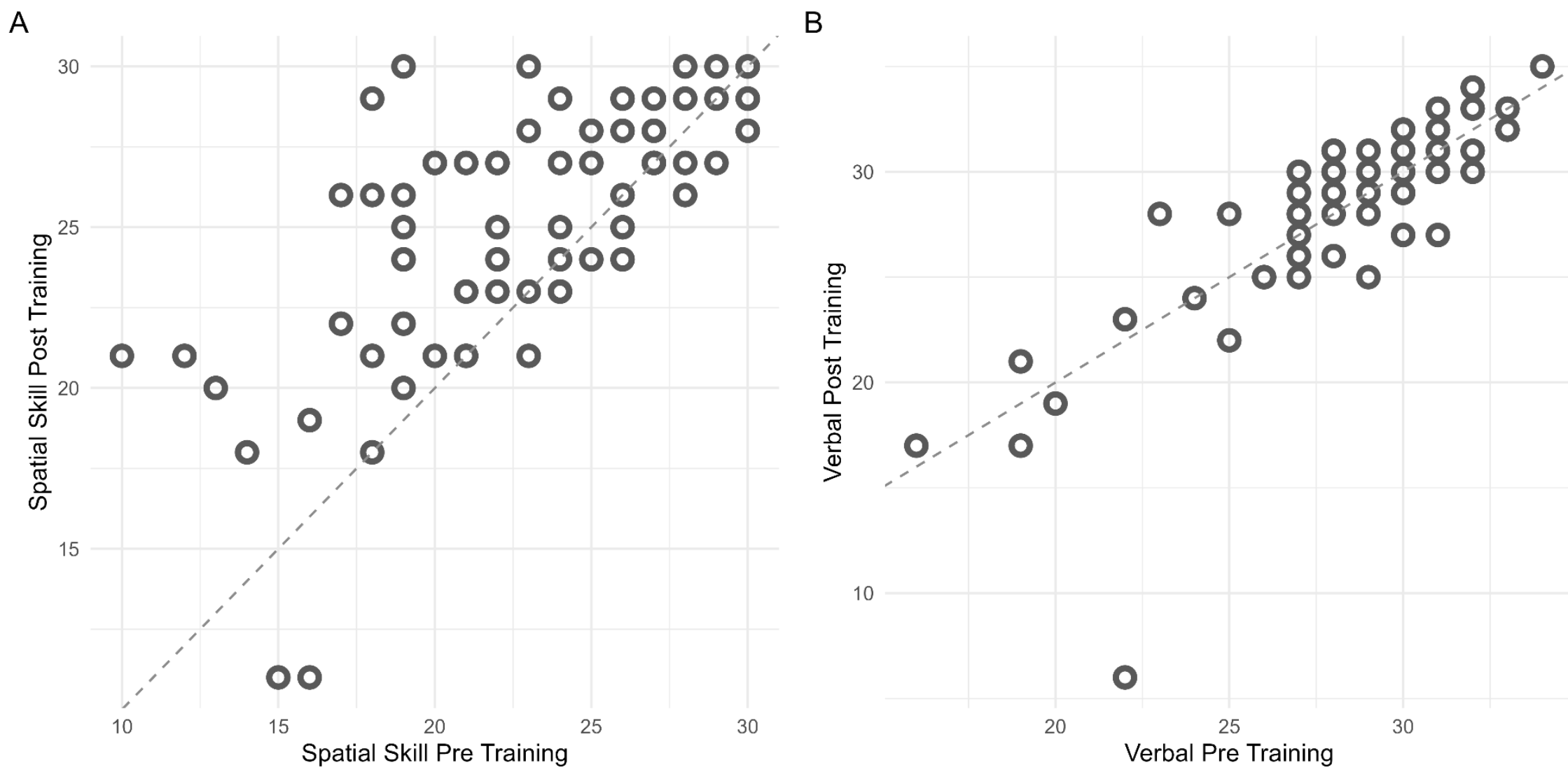

Fig. S5. Spatial and verbal behavioral abilities before spatial training (x-axis) and after spatial training (y-axis) showing the subjects in this study learned from the spatial visualization curriculum which was introduced during the in-scanner video lesson. (A) Spatial skills (PSVT:R scores) demonstrate substantial improvement after 9 weeks of spatial training (*N* = 71) and (B) KBIT:V scores (*N* = 62) reflecting verbal knowledge abilities as a control measurement (14).

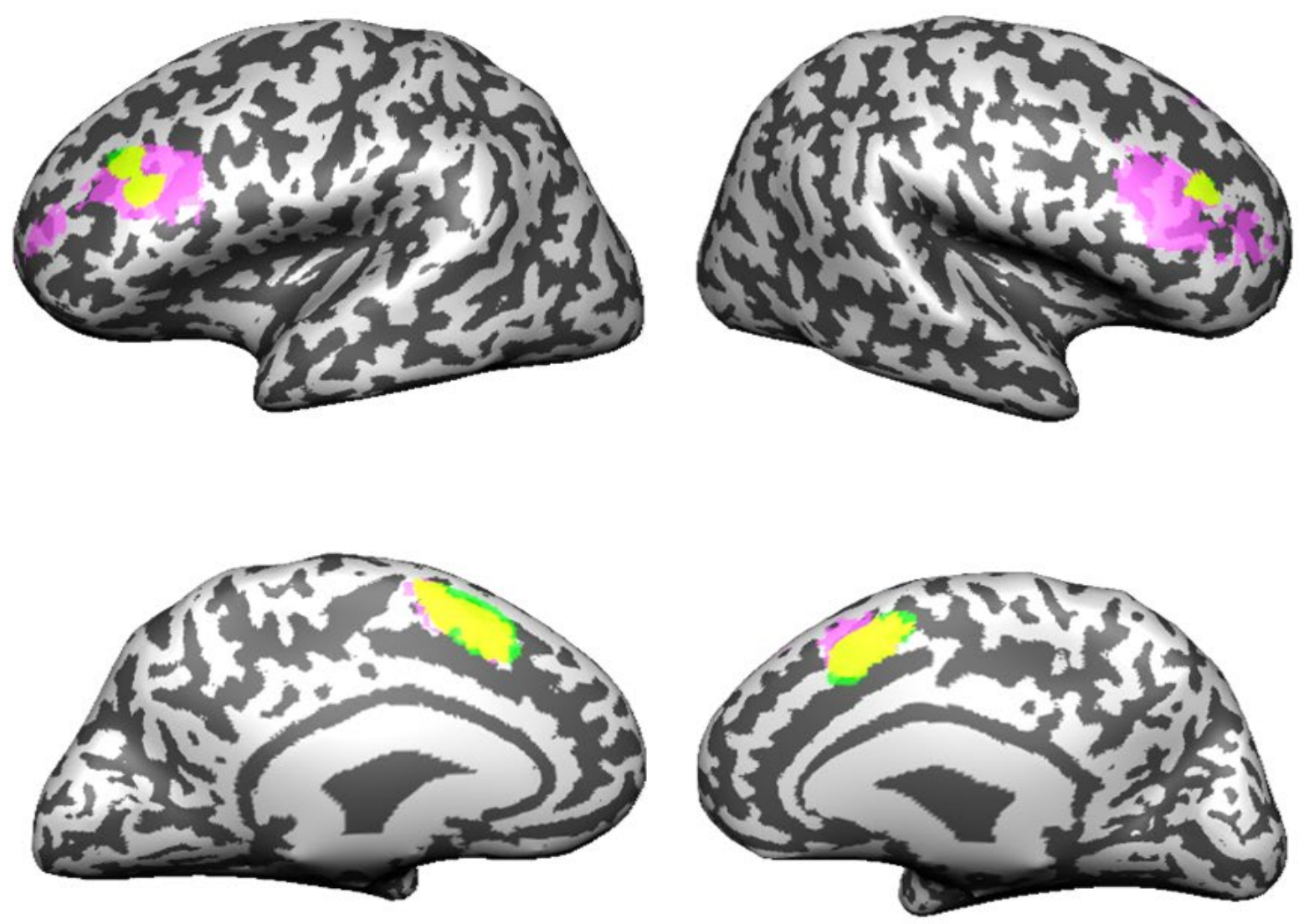

Fig. S6. Overlap between domain-general ROIs defined from the mental rotation localizer and domain-general frontal regions from the "Working Memory" Neurosynth meta-analysis (15). Prefrontal and cingulate regions identified from 1,091 "working memory" fMRI studies in Neurosynth are shown in pink. Domain-general ROIs derived from the mental rotation localizer used for primary analyses are shown overlaid in green (right DLPFC, left DLPFC, dorsal ACC). Overlapping voxels between the two maps are colored yellow, reinforcing domain-general network characterization. This map is an association-test z-map thresholded at FDR q = .01.

Tables

Table S1. Regions of Interest. Coordinates reported in Talairach space (2).

Region of Interest	X	Y	Z	Voxel Count	Network
L aIPS	-34	-42	42	15252	Math
L pIPS	-19	-65	46	23542	Math
L dPremotor	-25	-7	53	11494	Premotor
L vPremotor	-48	1	32	5527	Premotor
L DLPFC	-44	32	36	374	Domain General
dACC	-2	11	47	3901	Domain General
R aIPS	34	-39	41	11171	Math
R pIPS	19	-62	47	24461	Math
R dPremotor	25	-6	53	9321	Premotor
R vPremotor	50	3	32	517	Premotor
R DLPFC	39	40	36	247	Domain General
R med temp gyrus	54	-19	-6	734	Language
L med temp gyrus	-56	-28	-6	437	Language
L IFG	-50	10	4	602	Language
VWFA	-47	-55	-19	6213	Language

Table S2. Adjusted generalized variance inflation factor values ($GVIF^{(1/(2\times Df))}$) for instructional variable predictors included in the linear mixed-effects model predicting PSC. All adjusted GVIF values were below conventional thresholds for problematic collinearity.

	$GVIF^{(1/(2\times Df))}$
Network	1
Linguistic Density (z)	2.311814
Cognitive Load (z)	2.454491
Rotation Complexity (z)	2.433654
Network × Linguistic Density (z)	1.351957
Network × Cognitive Load (z)	1.435395
Network × Rotation Complexity (z)	1.42321

Movie S1 (separate file). Spatial visualization lesson. Developing Spatial Thinking curriculum (13).

SI References

1. Goebel, R., Esposito, F. Formisano, E. (2006). Analysis of functional image analysis contest (FIAC) data with Brainvoyager QX: From single-subject to cortically aligned group general linear model analysis and self-organizing group independent component analysis. *Human Brain Mapping*, 27(5), 392-401. https://doi.org/10.1002/hbm.20249.
2. Talairach, J., & Tournoux, P. (1988). Co-planar stereotaxic atlas of the human brain : 3-dimensional proportional system : an approach to cerebral imaging. G. Thieme ; New York : Thieme Medical Publishers.
3. R Core Team (2024). R: A Language and Environment for Statistical Computing. R Foundation for Statistical Computing, Vienna, Austria. <https://www.R-project.org/>.
4. Bates, D., Mächler, M., Bolker, B., & Walker, S. (2015). Fitting Linear Mixed-Effects Models Using lme4. *Journal of Statistical Software*, 67(1), 1–48. https://doi.org/10.18637/jss.v067.i01.
5. Kuznetsova, A., Brockhoff, P. B., & Christensen, R. H. B. (2017). lmerTest Package: Tests in Linear Mixed Effects Models. *Journal of Statistical Software*, 82(13), 1–26. https://doi.org/10.18637/jss.v082.i13.
6. Scrucca, L., Fraley, C., Murphy, T.B., & Raftery, A.E. (2023). Model-Based Clustering, Classification, and Density Estimation Using mclust in R (1st ed.). Chapman and Hall/CRC. https://doi.org/10.1201/9781003277965.
7. Wickham, H. (2016) ggplot2: Elegant Graphics for Data Analysis. Springer-Verlag, New York. https://doi.org/10.1007/978-3-319-24277-4_9.
8. Gu, Z., Eils, R., & Schlesner, M. (2016). Complex heatmaps reveal patterns and correlations in multidimensional genomic data. *Bioinformatics* (Oxford, England), 32(18), 2847–2849. https://doi.org/10.1093/bioinformatics/btw313.
9. Csardi, G., & Nepusz, T. (2006). The igraph software. *Complex syst*, *1695*(1).
10. Watson C.G. (2025). brainGraph: Graph Theory Analysis of Brain MRI Data. R package version 3.1.1. https://CRAN.R-project.org/package=brainGraph.
11. Guay, R. B. (1976). Purdue Spatial Visualization Test. West Lafayette, IN: Purdue Research Foundation.
12. Yoon, S. Y. (2011). *Psychometric properties of the Revised Purdue Spatial Visualization Tests: Visualization of Rotations (the Revised PSVT:R)* (Order No. 3480934). Available from ProQuest Dissertations & Theses Global. (904417099). https://www.proquest.com/dissertations-theses/psychometric-properties-revised-purdue-spatial/docview/904417099/se-2.
13. Sorby, S., Veurink, N., & Streiner, S. (2018). Does spatial skills instruction improve STEM outcomes? The answer is 'yes'. *Learning and Individual Differences*, 67, 209–222. https://doi.org/10.1016/j.lindif.2018.09.001.
14. Kaufman, A. S. (1990). Kaufman Brief Intelligence Test: KBIT. AGS, American Guidance Service.
15. Yarkoni, T., Poldrack, R. A., Nichols, T. E., Van Essen, D. C., & Wager, T. D. (2011). Large-scale automated synthesis of human functional neuroimaging data. *Nature Methods*, 8(8), 665–670. https://doi.org/10.1038/nmeth.1635.
